# Repurposed Biguanide Drugs in Glioblastoma Exert Antiproliferative Effects via the Inhibition of Intracellular Chloride Channel 1 Activity

**DOI:** 10.3389/fonc.2019.00135

**Published:** 2019-03-13

**Authors:** Federica Barbieri, Ivan Verduci, Valentina Carlini, Gianluigi Zona, Aldo Pagano, Michele Mazzanti, Tullio Florio

**Affiliations:** ^1^Sezione di Farmacologia, Dipartimento di Medicina Interna & Centro di Eccellenza per la Ricerca Biomedica, Università di Genoa, Genoa, Italy; ^2^Dipartimento di Biotecnologie Mediche e Medicina Traslazionale, Università degli Studi di Milano, Milan, Italy; ^3^Dipartimento di Neuroscienze, Riabilitazione, Oftalmologia, Genetica e Scienze Materno-Infantili, Università di Genoa, Genoa, Italy; ^4^IRCCS Ospedale Policlinico San Martino, Genoa, Italy; ^5^Dipartimento di Medicina Sperimentale, Università di Genoa, Genoa, Italy

**Keywords:** glioblastoma, cancer stem cells, CLIC1, biguanide, metformin

## Abstract

The lack of in-depth knowledge about the molecular determinants of glioblastoma (GBM) occurrence and progression, combined with few effective and BBB crossing-targeted compounds represents a major challenge for the discovery of novel and efficacious drugs for GBM. Among relevant molecular factors controlling the aggressive behavior of GBM, chloride intracellular channel 1 (CLIC1) represents an emerging prognostic and predictive biomarker, as well as a promising therapeutic target. CLIC1 is a metamorphic protein, co-existing as both soluble cytoplasmic and membrane-associated conformers, with the latter acting as chloride selective ion channel. CLIC1 is involved in several physiological cell functions and its abnormal expression triggers tumor development, favoring tumor cell proliferation, invasion, and metastasis. CLIC1 overexpression is associated with aggressive features of various human solid tumors, including GBM, in which its expression level is correlated with poor prognosis. Moreover, increasing evidence shows that modification of microglia ion channel activity, and CLIC1 in particular, contributes to the development of different neuropathological states and brain tumors. Intriguingly, CLIC1 is constitutively active within cancer stem cells (CSCs), while it seems less relevant for the survival of non-CSC GBM subpopulations and for normal cells. CSCs represent GBM development and progression driving force, being endowed with stem cell-like properties (self-renewal and differentiation), ability to survive therapies, to expand and differentiate, causing tumor recurrence. Downregulation of CLIC1 results in drastic inhibition of GBM CSC proliferation *in vitro* and *in vivo*, making the control of the activity this of channel a possible innovative pharmacological target. Recently, drugs belonging to the biguanide class (including metformin) were reported to selectively inhibit CLIC1 activity in CSCs, impairing their viability and invasiveness, but sparing normal stem cells, thus representing potential novel antitumor drugs with a safe toxicological profile. On these premises, we review the most recent insights into the biological role of CLIC1 as a potential selective pharmacological target in GBM. Moreover, we examine old and new drugs able to functionally target CLIC1 activity, discussing the challenges and potential development of CLIC1-targeted therapies.

## An Introduction to Cancer Stem Cells in Human Glioblastoma

Glioblastoma (GBM) is the most aggressive and prevalent primary brain cancer in adults, characterized by morphological, cellular, and molecular heterogeneity leading to invasive growth and resistance to therapy (1). Despite the use of aggressive multimodal treatments, GBM invariably recurs, and the median overall survival time of patients is extremely poor (~15 months after diagnosis) (2). The high drug resistance and recurrence rate of GBM is mainly ascribed to a sub-population of cancer stem cells (CSCs) within the tumor mass (3). GBM CSCs (GSCs) share functional features with neural stem cells, including self-renewal and multipotency, as well as the over-activation of biochemical signaling pathways (i.e., Sonic hedgehog, Akt, and Wnt/β-catenin). On the other hand, GSCs possess distinct genetic and epigenetic alterations which sustain their *in vivo* tumorigenic potential: through asymmetric division GSCs give rise to all the differentiated non-tumorigenic cells forming the bulk of the tumor mass, while their stem cell-like properties provide them with inherent resistance and evasion of apoptosis (4–6).

Phenotypically, GSCs are characterized by the expression of a combination of stem cell markers (e.g., CD133, Olig2, Sox2, Nanog), although different GSC populations exist, and a unique tumor-related phenotype has not been yet identified. Several proteins contribute to the maintenance of the stem-like phenotype, the aggressiveness, and the white matter invasiveness of GSCs, including CD44, sprouty2, Notch, tGLI1, and PrP (7–11). Moreover, the microenvironment in which GSCs develop is extremely complex, harboring non-neoplastic stromal cells, mesenchymal stem cells (MSCs), endothelial cells, immune cells, and other glial cell types, organized to compose the tumor niches (12). A dynamic and reciprocal crosstalk between GSCs, GBM bulk cells and the microenvironment cells occurs in the niches, via paracrine signals, mainly mediated by chemokine systems (for ex. CXCR4/7-CXCL12) (13) or direct cell-cell interactions. This microenvironment contributes tumor progression, invasion, angiogenesis, escape from immune surveillance, drug resistance, as well as to GSC maintenance, favoring the retaining of the stem-like properties (14, 15).

GSCs sustain neovascularization via the release of pro-angiogenic factors and vascular transdifferentiation (16), and are able to secrete cytokines inducing immune suppression (17, 18). Moreover, alteration of metabolic programs (i.e., the Warburg effect) drives the aggressive phenotype of GSCs providing them biosynthetic molecules useful for rapid growth (19).

Cytotoxic drugs, such as temozolomide, might favor a mutagenic selection of treatment-resistant GSC clones, further increasing GSC genetic heterogeneity, which represents a relevant mechanism for tumor recurrence (20). In addition, GSC and non-GSC populations retain dynamic interconversion through self-differentiation and de-differentiation, respectively (21, 22). Given the capacity of GSCs to generate all the different tumor cell populations composing the tumor mass, GSC targeting agents should be used in combination with existing therapies to arrest tumor growth and improve the clinical outcome.

Overall the complex nature of GSCs makes their eradication the main therapeutic goal for GBM, but a still unsolved challenge (23). In fact, conventional antitumor drugs spare GSCs, allowing tumor re-growth. Potential innovative strategies to eradicate GSCs from tumors are directed to: (i) impair specific pathways crucial for GSC survival and functioning (i.e., Notch, Wnt, Sonic hedgehog); (ii) targeting GSC perivascular or hypoxic niches; (iii) block metabolic and/or epigenetic modifications providing GSCs with stem-like properties. However, GSCs frequently activate multiple compensatory signaling pathways, change phenotype along tumor progression, displaying genetic heterogeneity, high plasticity and diversity of stemness markers, nullifying potential effective therapies (24). The identification of the distinctive GSC Achilles heel is an urgent goal for GBM treatment, since innovative therapeutic approaches identified for other cancer types left the survival of GBM patients practically unchanged over the past decades.

## Ion Channels in Cancer: CLIC1 Functional Expression and Therapeutic Potential

Ion channels are integral membrane proteins that form pores through which enable the passage of ions between cell compartments, regulating electrical excitation, cell proliferation, motility, survival, and maintaining tissue homeostasis. Structural defects or dysregulated functioning of ion channels play a pathogenic role in several human diseases including cancer. In particular, alterations of ion channel activity contribute to malignant transformation, inducing aberrant cell cycle rate, inability to activate the apoptotic program, and increased migration and invasion abilities (25). Genes encoding ion channels involved in oncogenic transformation (26) are differentially expressed in cancer and normal cells, in breast cancer (27), lung adenocarcinoma (28), and GBM (29).

While the role of plasma membrane channels has been extensively studied, less is known about intracellular ion channels. These molecules, inactive in the cytoplasm, are able to auto-insert into membranes where they act as functional integral ion channels, and have been recently recognized to regulate cell cycle, apoptosis, cell adhesion and motility (30). In this scenario, pharmacological modulation of intracellular ion channels would represent a potential innovative therapeutic option.

Among the ion channels whose aberrant expression and activity is relevant for neoplastic transformation, the chloride intracellular channel (CLIC) family recently gained particular attention. The six members of CLIC group (CLIC1-6), are present in both soluble and membrane-associated forms, displaying cell-specific expression and biological functions in mammalian tissues, not functioning as conventional chloride channels but possessing peculiar physiological roles in each different cell type (31). CLIC1 is the most widely expressed and studied channel of this family, in both physiological and pathological conditions, including brain functioning and cancer cell proliferation (32).

### Overview of the Mechanisms of CLIC1 Activation and Related Physiological Functions

CLIC1 is a metamorphic protein (33) able to switch from a soluble cytoplasmic conformation to a transmembrane isoform (tmCLIC1) (34). Thus, CLIC1 exists in three different states: a monomeric soluble form, an oxidized soluble dimeric intermediate form, and an integral membrane form. The soluble monomer contains a thioredoxin-like N-domain with a glutathione binding site. The formation of the dimer is stabilized through a disulfide bond which connects two conserved cysteine residues, Cys-24 and Cys-59, which are essential for channel assembly, as the mutation of each one of them prevents the channel formation (34). Cys-24 residue is also required for the protein redox regulation, rising the hypothesis that CLIC1 membrane insertion could be controlled by reactive oxygen species (ROS) signaling (35, 36). Membrane association implies the formation of oligomeric CLIC1 complexes (37).

The ability to form the channel pore was confirmed in artificial lipid bilayers by Littler et al. (38). Membrane-associated CLIC1 exposes the N-terminal region to the extracellular space, determining the ability to activate a selective chloride conductance. Both oxidizing conditions and changes in pH levels control CLIC1 membrane insertion. In fact, CLIC1 membrane insertion is not only dependent on the level of cellular oxidation, as suggested by the observation that the dimeric intermediate form is reversible under reducing conditions, but its assembling within lipid bilayers and channel activity are also dependent on pH, being minimal at pH 7 and reaching the maximum rate at ± 2 pH units (39, 40). Mutation of two histidine residues, His-74 and His-185, impairs CLIC1 pH sensitivity, preventing membrane insertion at acidic pH 5.5 (41).

### CLIC1 Signaling in Brain Function

CLIC1 is almost ubiquitously expressed in human tissues, including the central nervous system (CNS) where it is expressed in both excitable and non-excitable cells. CLIC1 is also present, in cytoplasmic conformation, in microglia, the brain intrinsic immune system (42, 43). Being chronic inflammation of the CNS during neurodegenerative disorders sustained by activated glia, tmCLIC1 was involved in the pathophysiology of Alzheimer's disease, considering that the neurodegenerative process implies an overproduction of ROS mediated by activated microglia (42, 44). This phenomenon can be reproduced *in vitro* stimulating microglial cells with amyloid β (Aβ) peptides: Aβ-activated microglia is characterized by high proliferative rate, production of large amount of ROS, and sustained by tmCLIC1 activity (42). Though tmCLIC1 rapidly increases in response to Aβ stimulation, it is rarely detectable in quiescent microglia cells (45). Indeed, CLIC1 downregulation in microglia by small interfering RNA or its inhibition using the channel blocker IAA94 and/or specific antibodies, prevents Aβ-induced neurotoxicity (45). Analogously, CLIC1 activity is a pre-requisite for ROS overproduction in β-amyloid-activated microglia (42). All together these findings indicate that tmCLIC1 plays a crucial role in the microglial inflammatory state characterizing the neurodegenerative processes, and support therapeutic targeting for neuroprotective strategies (44).

### CLIC1 in Cancer

**Figure 1:**
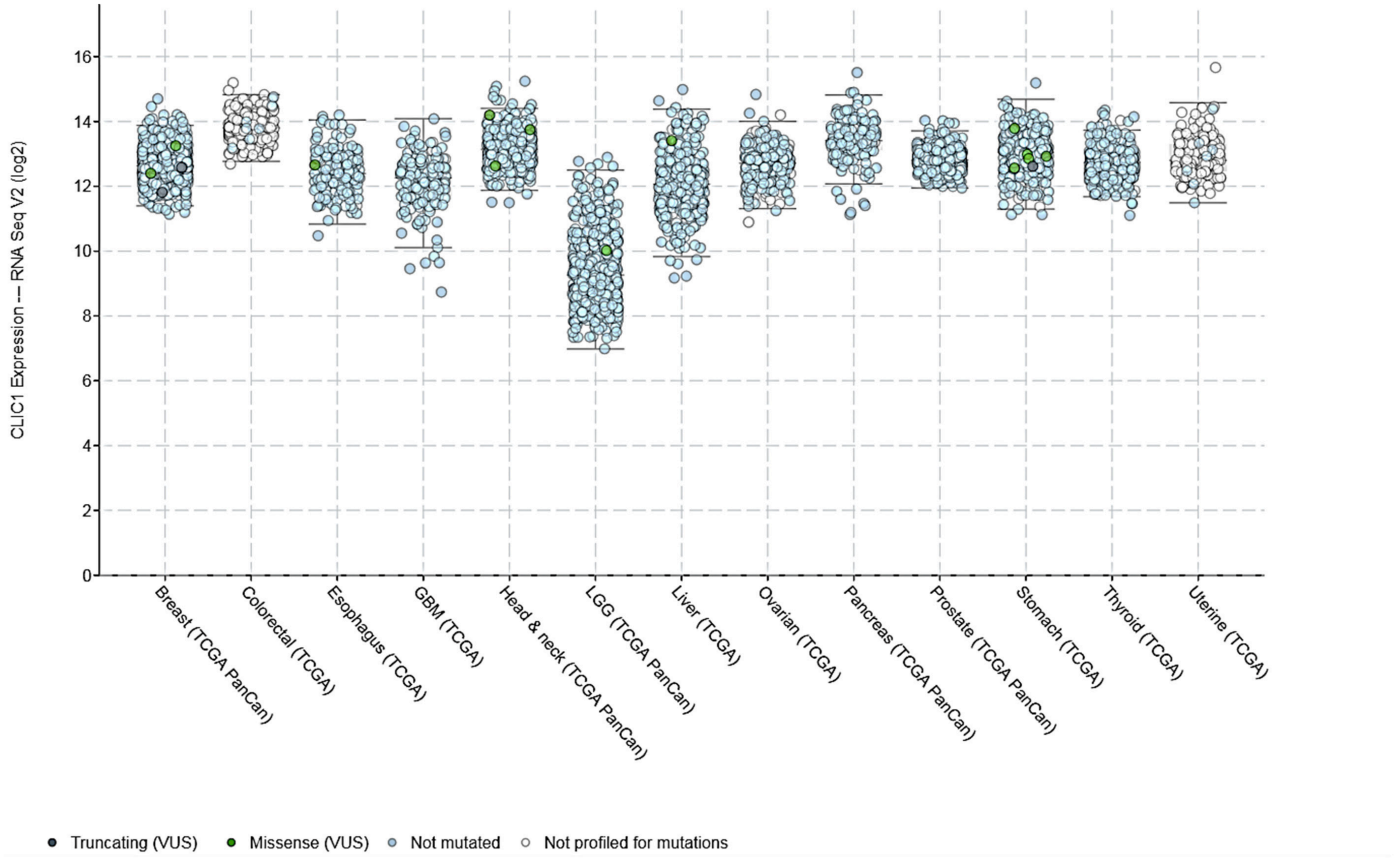
CLIC1 mRNA expression levels in various human carcinomas and mutations found according to the cBioPortal/TCGA datasets.

In the last years, growing evidence highlighted the role of CLIC1 as key factor in tumor development and progression. Working as a chloride channel, tmCLIC1 plays an essential role in tumorigenesis, controlling cell volume regulation (46), cell migration and invasion (47–49), and neoangiogenesis (50). CLIC1 is overexpressed in several human solid tumors, as compared to the surrounding normal tissue. For example, CLIC1 gene expression is significantly increased in bladder (51), *in situ* breast ductal (52) and ovarian (53) carcinomas, and it has been linked to oncogenic functions and poor prognosis in colorectal (48), gastric (49), hepatocellular (54), gallbladder (55), pancreatic (56), and lung carcinomas (57), and sarcomas (58). Bioinformatic analysis (cBioPortal/TCGA datasets) of CLIC1 mRNA levels in several human aggressive carcinomas (breast, colorectal, esophagus, liver, ovarian, stomach, prostate, thyroid, uterine, head & neck, and pancreas) shows that this channel is expressed at similar levels in all the different types of neoplasia, with a small increment only in colorectal, head & neck and pancreatic cancers (Figure 1). These data suggest the relevance of this channel in the development and progression of most malignant neoplasms. Moreover, CLIC1 gene is highly conserved among tumors in the various districts, with only 2% of patients carrying missense or non-sense mutations, clearly indicating that its role in tumorigenesis is more related to membrane localization and activity than to mutations.

The expression and function of several ion channels are altered in GBM cells, and, within chloride channels, changes in CLIC1 gene expression have been frequently detected (51). CLIC1 mRNA and protein levels are up-regulated in GBM as compared to normal brain parenchyma. The analysis of TCGA database identified a weak correlation with tumor stage, displaying lower expression in low grade gliomas than in GBM (Figure 1), suggesting a potential role for this channel in the malignant behavior of this tumor. Similarly, CLIC1 expression levels directly correlated to GBM aggressiveness in experimental models (30). Beyond expression levels, *in vitro* and preclinical *in vivo* studies analyzing CLIC1 channel function in malignant transformation and progression, shed new light on its biological and clinical significance in tumors. CLIC1 channel activity is involved in invasion and migration through ROS-mediated MAPK pathway in colon cancer cells (46, 48), and gastric cancer cells (49, 59). Also the metastatic process has been associated to CLIC1 functioning in gallbladder and hepatocellular carcinomas (55, 60).

The abundance of tmCLIC1 expression in cancer and its high activity in all the cells with sustained proliferation rate, raised the hypothesis of an oncogenic role of CLIC1 (43, 56).

### CLIC1 Role in Cell Cycle Progression of Cancer Cells

**Figure 2:**
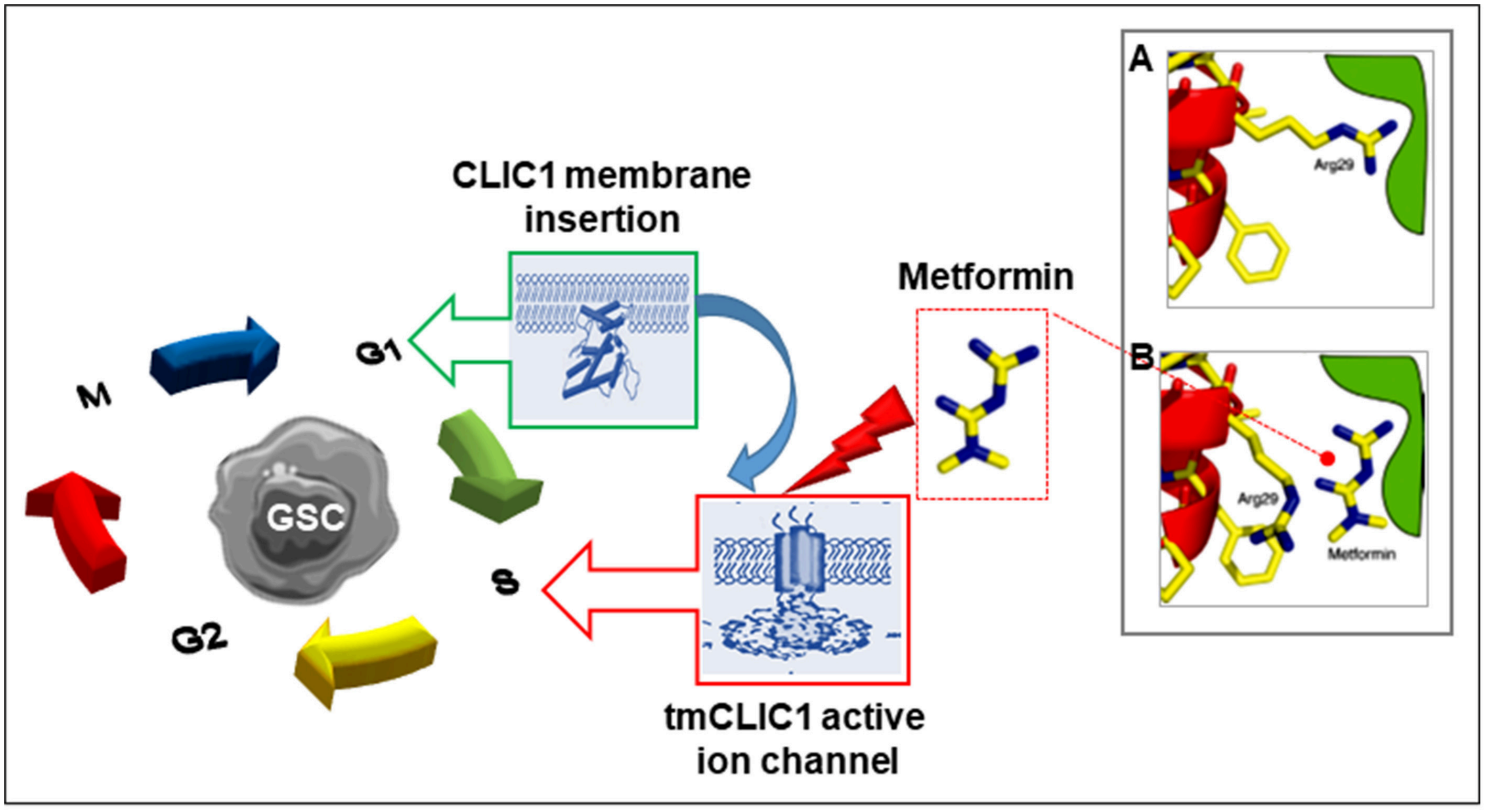
Scheme of the proposed mechanism by which metformin and other biguanides interacts with CLIC1 in glioblastoma stem cells. CLIC1 is a main regulator of GSC functioning once expressed into the plasma membrane and acting as chloride ion channel (tmCLIC1). CLIC1 activity promotes cell cycle progression and cell division. While in normal cells this functional expression transiently occurs during G1/S phase transition, it is constitutive in cancer cells leading to accelerated growth rate. Metformin (and other biguanides) directly interact with the extracellular portion of the active tmCLIC1, interfering with its activity and inhibiting cell cycle progression with high specificity toward GSCs, due to the high activity of CLIC1 in these cells. The insert shows a schematic representation of the putative molecular site of CLIC1 blockade by metformin: **(A)** In the closed state of CLIC1, the side chain of Arg29 makes an interaction that destabilizes the closed state. This facilitates the opening of the channel; **(B)** Metformin (and possibly other biguanides) interacts with the amino terminus of the channel, presumably in the vicinity of Arg29 side chain responsible for pore opening, stabilizing the closed state and blocking the channel activity [modified from Gritti et al. (68)].

Different chloride channels are involved in cell division and specifically in the regulation of cell cycle progression, showing a functional activity restricted to a specific cell cycle phase, the G1/S transition (61, 62). In physiological conditions, CLIC1 is mostly cytoplasmic and, upon an oxidative burst, it transiently inserts into the plasma membrane. However, after persistent oxidative stress and/or alkaline cytoplasmic pH, the integral membrane channel form becomes constitutive. Oxidation and cytoplasm alkalization are hallmarks of cancer cells (63, 64) and both conditions promote G1/S cell cycle progression (65, 66). Intriguingly, high ROS production, cytoplasm alkalization, and the subsequent G1/S transition occur in the same time-window in which CLIC1 is active as ion channel (43, 67). Indeed, tmCLIC1 functional expression undergoes a well-defined timing, as shown by electrophysiology measurements, demonstrating that chloride current increases along G1/S phase progression, reaching a peak just before G1/S transition (68) (Figure 2). Fluorescence intensity analysis of tmCLIC1 by TIRF microscopy supports these results, demonstrating a different localization of the protein during the different phases of the cell cycle (67). Moreover, the inhibition of CLIC1 activity with the specific blocker IAA94 (69), or using an antibody targeting the NH2 extracellular portion of the channel, induces the accumulation of cancer cells, including GSCs, in the G1 phase with a consequent delay of cell cycle progression (68). Elevated ROS levels and alkaline pH can result from the overexpression and/or hyperactivation of NADPH oxidase and Na^+^/H^+^ exchanger 1 (NHE1), respectively, and both NADPH oxidase and NHE1 activities are impaired by targeting CLIC1 function (67). In this scenario, functional expression and activation of tmCLIC1 trigger a feed-forward mechanism which involves the activity of NAPDH oxidase and NHE1 establishing a vicious loop which generates a cellular microenvironment that favors the abnormal proliferative rate of tumor cells (67).

### Role of CLIC1 in Cancer Stem Cell Proliferation

The relevance of CLIC1 in tumor biology, and for GBM in particular, is further supported by the observation that tmCLIC1 is highly expressed in patient-derived GSC sub-populations (30, 68). Moreover, CLIC1-mediated chloride current is higher in GSCs isolated from neurospheres and expressing stemness markers (nestin, Sox2, Olig2), than in the differentiated GBM cell counterpart (68). Inhibition of CLIC1 activity using IAA94 (69), anti-CLIC1 N-terminus antibodies, or CLIC1 downregulation using small interfering RNA, causes a reduction of self-renewal, proliferation and *in vivo* tumorigenic ability of patients-derived GSCs (30, 68, 70).

This evidence strengthens the idea that CLIC1 plays a critical role in the tumorigenic potential of GBM-derived stem/progenitor cells. The differential functional expression of CLIC1 between GSCs and their differentiated counterpart could represent a possible strategy to selectively recognize and hit the tumor stem cell subset. Thus, tmCLIC1 represents a potential ideal target for antineoplastic treatments, also as chemo-sensitizing approach which, hitting CSC subpopulations, may increase tumor responsiveness to conventional anticancer therapies.

## Therapeutic Potential OF CLIC1 Pharmacological Targeting in Glioblastoma

### Rationale for Targeting CLIC1

To date, GBM represents the biggest challenge for cancer therapy. The main reason for the failure of GBM treatments is represented by tumor occurrence in one of the most critical area of human body, physically shielded by the skull and pharmacologically isolated by the BBB. Although GBM, as every tumor, represents a detriment from a clinical point of view, cancer cells may be considered an evolutionary successful model, being able to dynamically adapt the changing microenvironment and reprogram their own physiology setting in a new steady state. Tumor cells are in a chronic hyper-activated (allostatic) state (71) that supports their abnormal proliferative rate. A novel strategy for GBM treatment could be to hit one or more components that promote the assessment of the chronic allostatic state, restoring the physiological homeostasis (67). Several proteins, including NADPH oxidase and NHE1 exchanger, involved in the establishment of the allostatic condition (72, 73) are crucial for survival of both cancerous and normal cells, therefore limiting the possibility of their pharmacologic or genetic targeting. In this scenario, the peculiar ability of CLIC1 to change its functional localization depending on the activation state of the cells could be a compelling strategy to impair tumor cell proliferation and/or survival with a higher efficacy in CSCs (67). The possibility to selectively hit the CSC fraction could be instrumental for a more efficient activity of standard antineoplastic drugs, also considering that CLIC1 inhibition-dependent delay of G1/S phase transition might also favor microglia activity toward tumor cells, and potentiate conventional cytotoxic therapies.

Cellular and molecular steps through which ion channels, including CLIC1, support malignant cell phenotype and specifically CSC features (enhanced survival and proliferation rate, self-renewal, migration ability, and resistance to apoptosis and chemo- or radio-therapy) are still not completely defined. However, a growing bulk of evidence is currently available to be exploited in pre-clinical investigation or in medicinal chemistry studies for the identification of novel compounds able to target ion channels involved in cancer cell proliferation.

CLIC1 displays several peculiar features which render this channel an ideal pharmacological target in cancer cells. First, CLIC1 is overexpressed in several cancer types as compared to non-cancer cell counterparts; second, its activity is pivotal for cancer cell functioning; third, although in physiological conditions it is ubiquitously expressed, its chloride channel activity, absolutely dependent on its membrane insertion, is constitutive only in tumor cells and, in particular, in the CSC compartment (32). In fact, the translocation of CLIC1 to the membrane is reversible and the channel activity is transient in normal cells, with only few channels active at a given time. On the contrary, in cancer cells the specific intracellular microenvironment generated by the high production of ROS and low pH levels (74, 75) enhances CLIC1 functional expression promoting its constitutive membrane localization as an active ion channel (67). Given that tmCLIC1 is largely more abundant in GSCs than in healthy tissues (30), the possibility to specifically hit the transmembrane isoform could be a promising novel therapeutic approach for GBM. Indeed, a successful therapy should slow-down the proliferation of GBM cells, preventing relapses by inhibiting GSCs with the minimum possible systemic toxicity. However, IAA94, the only known compound able to block CLIC1 activity *in vitro* (69), can't be used as a potential drug for GBM due to its off-target toxicity *in vivo*.

Importantly, recent studies identified the well-known antidiabetic drug metformin as a compound able to impair tmCLIC1 activity (68) (Figure 2). Metformin is a generally very well tolerated type 2 diabetes (T2D) first line drug, which displays antineoplastic effects, although the molecular mechanism at the basis of this effect is still debated. Thus, understanding at a molecular level how metformin interferes with cancer cell proliferation and the role of CLIC1 in such effect might improve its repositioning as antitumor agent or, alternatively, allow the development of structural-related molecules showing higher efficacy and potency against tmCLIC1.

## Development of Pharmacological Tools to Target CLIC1 Activity to Counteract Glioblastoma Cancer Stem Cell Tumorigenesis

The low clinical outcome of the available therapeutic approaches for GBM urges the identification of novel molecular targets and new molecules able to hit them. In this respect, as detailed in the previous paragraphs, CLIC1 represents a potential ideal candidate, for its relevance in GSC biology and its functional irrelevance in normal cells. If these premises are confirmed, a selective CLIC1 inhibitor should have high efficacy against tumor cells and low toxicity on the normal cell counterparts.

Unfortunately, the introduction of new molecular entities in clinics is becoming more and more difficult, due to the outraged increased costs of development and the tighter regulatory rules. Therefore, in the last years the approval of novel chemotherapeutics, with the only exception of biologicals, faces a significant slow-down. Drug repositioning, a strategy based on the identification of new disease indications and/or molecular targets for existing compounds, represents a drug discovery strategy which bypasses all the preclinical and early phase clinical trials and allows a faster, more efficient and less expensive way to bring molecules from bench to bedside. This is especially true if the studied drug has already proven good safety and tolerability profile in humans (76). Interestingly, a CLIC1 inhibitory activity was reported in some Chinese traditional medicine molecules, identified by bioinformatic strategies (77), although most attention has been dedicated to the effects of metformin a biguanide antidiabetic drug. In particular, it was shown that metformin is a powerful CLIC1 inhibitor in GSCs (78), and its repositioning as GBM drug could have a significant impact for the treatment of these patients. However, metformin represents the most studied repurposed drug in oncology in almost all human tumors and several intracellular mechanisms were proposed to mediate these effects. Thus, to show that CLIC1 inhibition is a primary target for this drug in GBM a pharmacological class effect should be demonstrated, showing that also structurally related drugs (i.e., containing a biguanide moiety) have the same biochemical mechanism (CLIC1 inhibition) and clinical effects (antitumor activity).

In the next sections we will discuss the general pharmacology of metformin (and of other biguanides), the evidence of their antiproliferative effects, and data showing that CLIC1 is one of the main molecular targets involved in the inhibition of GSC proliferation and invasiveness induced by this class of drugs, highlighting the pros and cons of their possible use for treatment of GBM.

### Pharmacology of Biguanides

**Figure 3:**
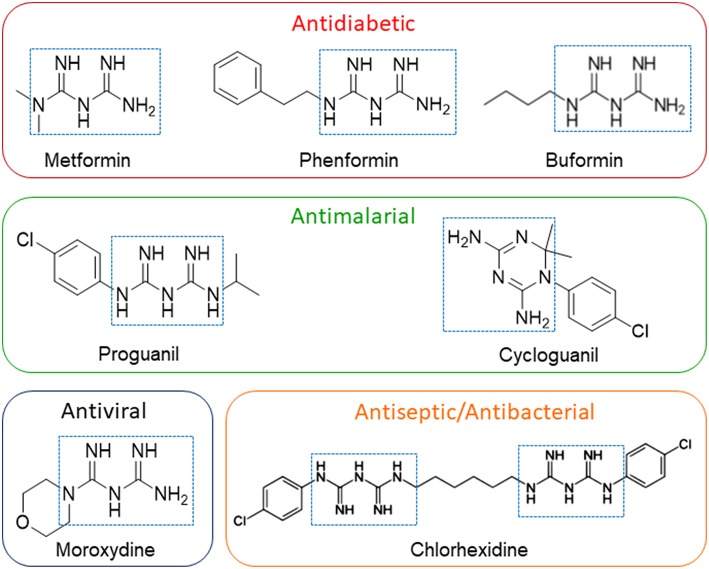
Structures of drugs possessing a biguanide moiety as pharmacophore. The presence of the biguanide moiety in clinically-relevant compounds, highlighting their actual clinical applications, is evidenced by the dotted squares. Figure includes compounds affecting CLIC1 activity to inhibit GSC proliferation and self-renewal.

Biguanides are a class of drugs whose functional group consists of two guanidines linked by a common nitrogen (Figure 3); biguanides have a broad range of medical indications spanning from the first line pharmacological approach for T2D, by metformin and its derivatives phenformin and buformin (although the latter compounds are no longer used in therapy), to antimalarial prophylaxis and therapy by proguanil, and antiviral and antimicrobial activity by moroxydine, chlorophenylbiguanide, and chlorhexidine.

Original interest in biguanides derived from their potential antimalarial effects, particularly by proguanil the first compound of this class used in 1950, and still a current antimalarial drug. Proguanil is a synthetic arylbiguanide acting as oral prodrug and is considered the safest antimalarial compound; *in vivo* it is metabolized to the active derivative cycloguanil, which contains a cyclized biguanide moiety and acts as dihydrofolate reductase and folate synthesis inhibitor within malaria parasites (79). Proguanil is used for both malaria prophylaxis and treatment, in combination with atovaquone or cloroquine (80). The observation that this drug may cause hypoglycemia as side-effect, triggered the development of the dimethylbiguanide metformin (81).

Metformin was licensed as anti-diabetic agent in the UK in 1958, but only in 1995 in the USA, due to concerns about lactic acidosis and cardiac mortality, which, however, are now considered as very rare occurrences. Among the different biguanides introduced for diabetes therapy in late 1950s, metformin shows the better safety profile and tolerability (82). Two other biguanides, phenformin (phenethyl biguanide) and buformin (N-butyl biguaninde), although more potent than metformin as hypoglycemizing agents, were discontinued in 1970s due to the same adverse events (lactic acidosis and cardiac mortality) but occurring at higher rate than observed with metformin (83). In T2D patients, glucose-lowering effect of metformin is attributed to the reduction of hepatic glycogenolysis and gluconeogenesis, enhancement of insulin receptor tyrosine kinase activity, improvement of insulin sensitivity, and reduction of enteric glucose absorption (84). Metformin also induces peripheral glucose uptake, increasing glucose transporter GLUT4 activity, and glycogen synthesis, stimulates glucagon-like-peptide-1 (GLP-1) release, and reduces lipolysis and triglyceride levels.

Moroxydine, is another heterocyclic biguanide proposed in the 1950s as anti-influenza agent. Moroxydine exhibits anti-viral activity against RNA and DNA viruses, and was originally used for prophylaxis or therapy of viral infections. Moroxydine has negligible side effects, but very little information exists on its mechanism of action (85). Despite its favorable pharmacological profile, moroxydine has been scanty investigated, and only recently it has gained new interest as potential anti-hepatitis C agent (86).

### Repositioning of Metformin and Other Biguanides as Antitumor Agents

#### Repositioning of Metformin as Anti-tumor Agent

On the basis of several epidemiological and preclinical observations, several biguanides have been proposed to possess anti-neoplastic activity; to date, metformin is the most promising application of repositioning of a non-oncological drug as anti-cancer agent. Epidemiologic studies suggested a correlation between chronic use of metformin in T2D patients and the reduction of incidence and related mortality of several solid tumors, when compared to T2D patients treated with other classes of hypoglycemic drugs (87–89). These observations triggered a series of pre-clinical and clinical investigations in several tumor types to detail the antitumor mechanism of action of metformin and its potential efficacy as adjuvant agent in clinics (90, 91). In diabetic patients, metformin use was correlated to a reduced risk of development of different cancer types, including pancreatic cancer (92), and lung and hepatocellular carcinomas also increasing survival time (93–95). However, to date non-univocal results were reported in the many studies published. Some meta-analyses confirmed a significant association between metformin use and lower incidence of pancreatic, liver, renal, endometrial, prostate, breast, colorectal, and ovarian carcinomas, while no correlation was found in other studies (96–103). Metformin use in T2D patients with HER2-positive breast cancer was associated with a better outcome (104) and a meta-analysis in breast cancer patients reported a significant association between metformin therapy and the reduction of all-cause mortality without observing a reduction of breast cancer incidence in these subjects (105).

#### Repositioning of Other Biguanides

Although less investigated than metformin, also other biguanides were shown to possess anti-cancer activity. Phenformin exerts antitumor activity in preclinical models *in vitro* and *in vivo*, using ovarian cancer (106) NSCLC (107), and hepatocellular carcinoma (108) cells, and pancreatic cancer patient-derived xenografts (109); moreover, phenformin was also reported to selectively affect the CSC compartment (110). Other studies showed that the antitumor activity of phenformin in mammary cancer was dependent on the inhibition of angiogenesis, apoptosis, and epithelial-mesenchymal transition (EMT) (111–113). The anti-cancer activity of buformin in rat mammary breast cancer carcinogenesis was also reported (114, 115).

However, compared to the other biguanides, the evidence of a potential broad antitumor activity, associated with the overall safety and low cost, has opened a new horizon for repurposing of metformin in oncology (87, 116, 117).

#### Repurposing of Biguanides for Targeting of CSCs

Several preclinical studies reported that metformin is effective against CSC subpopulations, the key target for all antitumor pharmacological approaches, at odd with most conventional anti-neoplastic drugs which have little or no effect on CSCs (118). Selective anti-cancer properties of metformin have been described in CSC-like cultures derived from colorectal (119), gastric (120), breast (121, 122), prostate (123), pancreatic (124, 125), and ovarian (126) carcinomas and osteosarcoma (127, 128). Metformin effects on CSCs include the impairment of self-renewal and survival, decreased expression of stemness markers, the slow-down of cell cycle progression and inhibition of invasiveness. Moreover, a chemo-sensitizing activity of metformin, helping to overcome refractory CSCs to radiotherapy (129), and chemotherapeutic agents (130) has been also described. As far as GBM, metformin was reported to synergize with temozolomide (131) and reduce the acquired resistance to this alkylating agent (132).

While most of the human tumors, at least at preclinical level, are affected by metformin, this drug is almost completely harmless for normal cells. The low toxicity observed in T2D patients after chronic treatment already suggested this eventuality, but it was directly demonstrated by *in vitro* experiments, in which metformin concentrations able to reduce CSC viability were ineffective in normal cells, including MSCs (68, 70, 133). These data clearly suggest that a tumor-specific target should mediate metformin antitumor effects.

To date, mainly pharmaco-epidemiologic and preclinical data were at the basis of the assumption that metformin may be useful in cancer prevention or treatment. However, hundreds of clinical trials are in progress to validate this hypothesis (see www.clinicaltrials.gov). Translation from retrospective to prospective trials however, is not easy-going also in light of several biases often present in retrospective studies (134). Some preoperative or neo-adjuvant window of opportunity studies reported a decrease in the expression of Ki-67, a marker of cell proliferation, after metformin treatment in breast, prostate, and endometrial cancers (135–137), although another study found no effects in breast cancer (138). An unpublished study (NCT01620593) found a significant decrease of prostate-specific antigen (PSA) after treatment of prostate cancer patients with metformin, while, in ovarian cancer (NCT01579812) no changes in progression-free and overall survival were reported (www.clinicaltrials.gov). Only few prospective studies have been to date published, reporting that metformin provided benefit in patients in colorectal adenoma and, in association with paclitaxel, in non-small cell lung cancer patients (139, 140).

Phenformin has been evaluated in a clinical trial (NCT03026517) in combination with dabrafenib and trametinib (RAF and MEK inhibitors, respectively) in patients with BRAF-mutated melanoma, but, till now, no results are available.

Overall the available literature data about the clinical antitumor efficacy of metformin are not conclusive, possibly due to the heterogeneous composition of patient cohorts, the study design, pharmacokinetics and posology discrepancies, as well as variable responses in different cancer types (141). Thus, repositioning of metformin and, potentially, other biguanide derivatives, in oncology is still a controversial topic, and results from clinical trials that are going to be concluded in the next years in different cancer types, mainly investigating the adjuvant efficacy of metformin in association with chemo- and radio-therapy, will provide a clearer picture of its clinical impact.

Notwithstanding these unsolved problems, a huge amount of data has been produced to detail the molecular mechanism(s) of the antiproliferative activity of metformin.

### Molecular Mechanisms of Metformin Antitumor Effect

Although numerous experimental studies analyzed the antiproliferative, pro-apoptotic, and anti-invasive activity of metformin, at present, the exact molecular mechanisms through which this drug exerts its antitumor activity is only partially known. In fact, most of the possible intracellular pathways involved in tumor cell proliferation have been reported to be affected by metformin treatment in different cancers. Consequently, not only metformin seems to not display tumor specificity but also its activity seems to involve a wide plethora of intracellular signaling pathways.

The classical intracellular pathway proposed as molecular target for metformin antitumor effects has been derived by the mechanism activated in the liver to control glucose release (142). Metformin affects the energetic balance interfering with the complex I enzymes within mitochondrial respiration, reducing ATP content and the ATP/ADP ratio (143). This alteration, altogether with a direct regulation via liver kinase B1 (LKB1), causes the activation of the cellular energy sensor AMP-activated protein kinase (AMPK), which in turn leads to the inhibition of mTOR (144, 145), a kinase acting as crucial mediator of tumor cell metabolism (146). AMPK, activated after metformin treatment, was reported to directly phosphorylate PD-L1 causing its endoplasmic reticulum (ER) accumulation and ER-associated protein degradation. In fact, breast cancers from metformin-treated patients exhibit reduced PD-L1 levels, which enhances cytotoxic cell immunity against cancer cells (147). In addition, metformin, lowering the plasma levels of insulin and insulin-like growth factors, might indirectly inhibit the PI3K/Akt/mTOR pathway (148). AMPK activation following metformin treatment has been described in several human cancer cell types including breast (149, 150), endometrial (151), ovarian (152), pancreatic (153, 154), lung (155), prostate (123), head and neck (156), and colon carcinomas (157), often correlating with antiproliferative effects.

However, AMPK-independent pathways have gained increasing attention. Metformin was reported to directly inhibit mTOR signaling by inactivating Rag GTPases (158), or inducing cell cycle arrest through REDD1, a negative regulator of mTOR (159). Furthermore, several other intracellular effectors were reported to be modulated by metformin treatment to reduce cell proliferation, including, among others, the VEGF/PI3K/Akt pathway (160) in prostate cancer cells, Sonic hedgehog (Shh) signaling pathway in gastric cancer cells (161), inactivation of p38 MAPK and activation of ERK3 (both effects leading to inhibition of mTORC1, in which AMPK was only partially involved) in intrahepatic cholangiocarcinoma cells (162), reversal of the activation of ERK1/2 in ovarian cancer cells (163), inhibition of CLIC1 in gallbladder cancer cells (164); metformin also counterbalanced the overactivation of Notch1/Hes1 signaling observed in colorectal cancer patients (165), and induced apoptosis via the up-regulation of adenosine A1 receptor in human colorectal cancer cells (166). Other putative mechanisms of metformin anti-tumor activity involve the reduced RANKL (167) or caveolin 1 (168) expression in breast CSCs, and HIF-1α gene expression in oral squamous cell carcinoma cell lines, which caused inhibition of cell proliferation and migration (169). Moreover, metformin antiproliferative activity was also ascribed to enhanced autophagy in cancer cells, which causes cell cycle arrest or apoptosis (170, 171), and the modulation of miRNA activity (172, 173).

In addition, metformin downregulates ROS production of through inhibition of mitochondrial complex I (174, 175), and possesses anti-inflammatory and immunomodulatory activity, affecting energy metabolism of immune cells and stimulating CD8^+^ tumor infiltrating lymphocytes leading to a cytotoxic response against cancer cells (176); moreover, metformin enhances immune response *in vivo* in mouse melanoma model (177), and inhibits NF-κB nuclear localization and Stat3 activity in breast cancer CSCs (178).

Metformin disrupts TGFβ-mediated oncogenesis and invasiveness (179) either by direct binding (180) or by blocking autocrine TGFβ1 signaling (181). TGFβ1-dependent metastasization and invasive effects are mainly mediated by epithelial-to-mesenchymal transition (EMT). In this context metformin acts as EMT suppressor in different epithelial tumors (e.g., melanoma, colon, breast, lung, prostate, and thyroid cancer cells) (182–185). In prostate cancer, metformin represses EMT and metastasis by targeting the COX2/PGE2/STAT3 axis (186), while in breast cancer the AKT/mTOR/ZEB1 pathway was involved (187). Metformin also directly affects cancer cell metabolism interfering with glycolysis and the tricarboxylic acid cycle, decreases the production of ATP, NADH-linked respiration in cells and mitochondria, and the aspartate biosynthesis (188, 189), while induces indirect antiproliferative effects reducing hormones, cytokines and growth factor production (144, 190, 191).

Altogether, these preclinical studies, reporting metformin ability to modulate multiple, apparently unrelated mechanisms, strongly support its antitumor activity. However, the unexpected and unprecedented high number of different intracellular mechanisms regulated by a single drug in such different tumor cell types, suggests that most of these intracellular pathways could be indirectly modulated, being downstream from a common tumor-specific target directly affected by metformin.

However, it is worth to note that several unsolved issues are present in these studies. First, metformin concentrations used to cause antitumor effects in all *in vitro* studies here reported, largely exceed those obtained by the antidiabetic doses, and are difficult to be reached in patients. A second issue puzzling the anti-cancer use of metformin is its hydrophilic nature which limits its passive diffusion into cells (192), making necessary organic cationic transporters (OCT1, OCT2, and OCT3) for its internalization within cells (193, 194) and to cross the blood-brain barrier (195). The overexpression of these transporters is considered at the basis of the observation that metformin concentration in tissues is much higher than in plasma, and in tumors higher than in normal cells. Intratumor accumulation of metformin, induced by OCTs, has been involved in the direct antineoplastic activity of this biguanide (196). For example, in mammary tumor-bearing rats and in ovarian tumor biopsies form metformin-treated patients, metformin effects were dependent on high intratumor concentrations, which in the mammary cancer model were related to OCT2 expression (197, 198). Thus, it was suggested the possibility to potentiate metformin antiproliferative activity, obtaining clinically relevant concentrations due to the specific drug accumulation within tumors. This opportunity was demonstrated using pharmaceutic preparations and routes of administration different from the oral way (i.e., subcutaneous) allowing a topical tumor treatment (199–201).

### Potential Role of Metformin and Other Biguanides as Antiproliferative Agents for Glioblastoma Stem Cells

Although *in vitro* studies reported the antiproliferative and proapoptotic efficacy of several drugs on GSC cultures (202–204), the same activity in patients was never reported. Thus, the lack of effective antitumoral drugs for GBM patients, pushed the testing of metformin repositioning in *in vitro* and *in vivo* GBM models (78).

**Figure 4:**
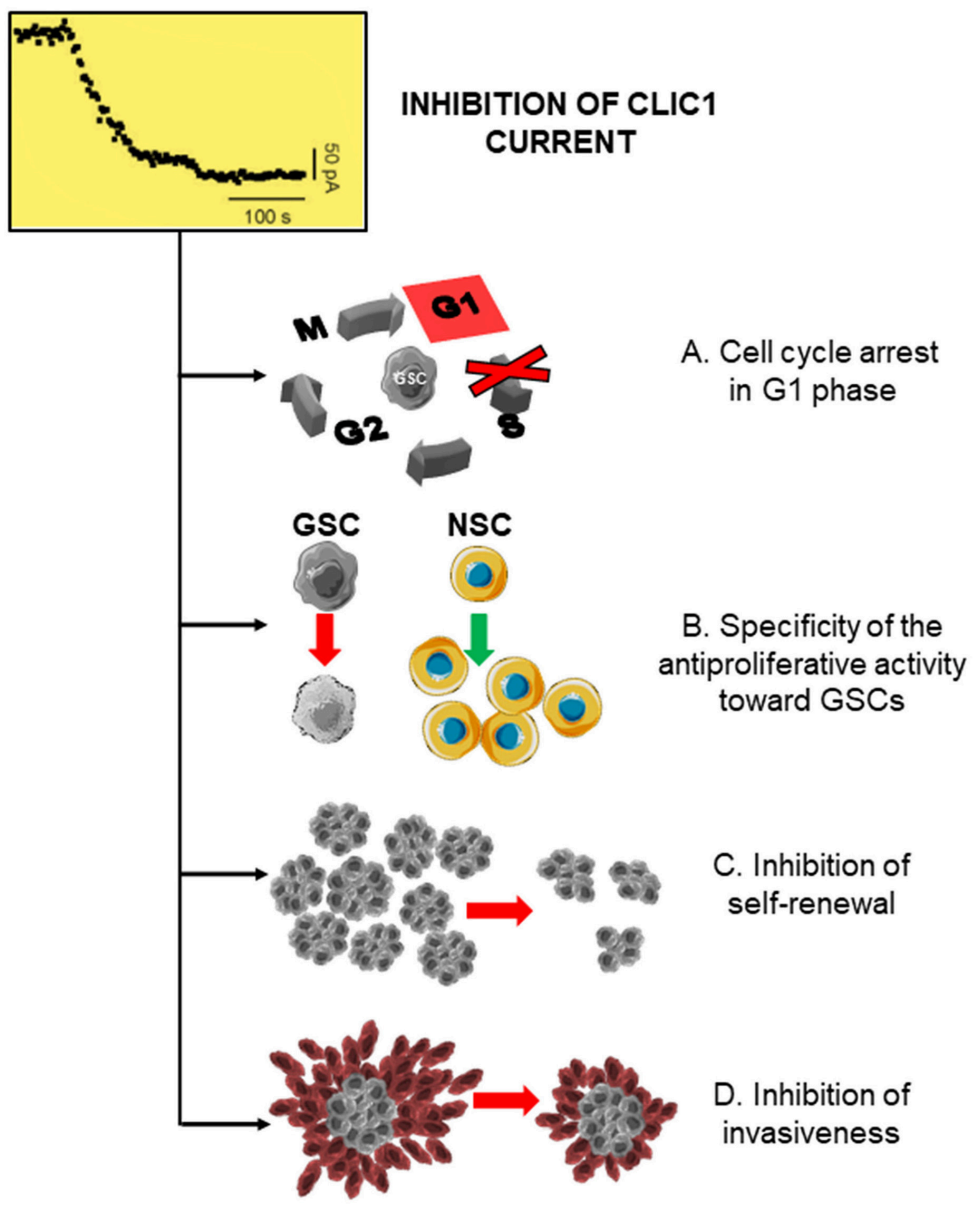
CLIC1 inhibition leads to the inhibition of glioblastoma stem cell proliferation, self-renewal, and invasiveness. Inhibition of CLIC1 activity by metformin and other biguanides causing a decrease in the chloride current induces several inhibitory effects in GSCs, including: **(A)** cell cycle arrest and cell accumulation in G1 phase, acting **(B)** selectively in GSCs, while sparing normal stem cells (NSC); **(C)** impairment of GSC self-renewal ability; and **(D)** inhibition of the invasive behavior.

Metformin reduces survival and proliferation rate not only of GBM cell lines (131, 205–207) but also of patient-derived GSC cultures (68, 70, 208–211) suggesting its efficacy to impair mechanisms involved in cancer cell stemness. This effect is time-dependent, since prolonged treatment caused significant antiproliferative effects also for relatively low concentrations (68). Importantly, metformin interference with GSC activity was further supported by the observation that, beside proliferation, several distinctive stemness features were also impaired in metformin-treated cultures, including self-renewal ability, as shown by colony-forming and spherogenesis assays (70, 132, 210, 211), migration and invasiveness (132, 210, 211) (Figure 4), and EMT (187), which is activated in GSCs to sustain GBM aggressiveness (212).

Metformin was also tested in combination therapy with classical anti-cancer drugs, mainly the alkylating agent temozolomide, the GBM standard of care. *In vitro* and *in vivo* studies describe a potential synergism between the antiproliferative effects of metformin and temozolomide in GBM cell lines and GSCs (131, 132, 206, 207, 213). Moreover, a strong synergism between the antitumor effects of metformin and *in vitro* cell irradiation (5Gy), in the presence or absence of temozolomide, was also reported in GBM U87, U251, LN18, and SF767 cells (206).

Also in GBM several intracellular mechanisms mediating the antiproliferative and anti-invasive activity of metformin were reported. These include the inhibition of STAT3 (214) and Akt (70), the induction of apoptosis by increasing Bax/Bcl-2 ratio, reduced ROS production when co administered with temozolomide (213), or the downregulation of AKT-mTOR signaling pathway (207). Although some studies proposed an AMPK-dependent mechanism for the antitumor activity of metformin (208, 209, 215, 216) in other studies the inhibition of proliferation and self-renewal occurred in the absence of AMPK activation (70). Moreover, comparing the effects of metformin with a “pure” AMPK activator, the peptide A769662, which was unable to inhibit mTOR and GBM cell proliferation, it was shown that metformin suppresses GBM proliferation enhancing PRAS40–RAPTOR association to inhibit mTOR, independently of AMPK (217). Although this issue is still debated, recent data seem to confirm that the activation of AMPK and the inhibition of mTOR are not the main targets in GBM. In fact, on one hand a randomized phase II study assessing the efficacy of everolimus in combination with chemoradiation showed that mTOR inhibition does not improve GBM patients PFS (218), and, on the other, AMPK was shown to be chronically activated under cancer-associated stress conditions, to increase proliferation and survival. Moreover, AMPK inhibition reduces viability of patient-derived GBM stem cells (GSCs) (219), clearly indicating that, at least in GBM, AMPK activation cannot justify metformin antiproliferative effects and different molecular targets have to be found.

Furthermore, several studies showed that metformin activity was selectively directed against GSCs rather than differentiated glioma cells (68, 70, 211), indicating that a CSC-specific target mediates its activity.

*In vivo*, metformin significantly impairs GBM growth either after subcutaneous (206–208, 217) or intracranial grafting (210, 213, 220) in immunocompromised mice. These effects were obtained mainly after i.p. injection, although in one study (207) metformin was administered *per os* by gavage. In the cited studies, metformin induced a slow-down in tumor growth and prolonged mice survival, mainly acting in synergy with temozolomide or 2-deoxyglucose, inducing a significant effect also in temozolomide-resistant cells (213). However, it is worth to note that most studies were carried out on human GBM cell lines (mainly U87, U251) and only in few cases patient-derived GSCs were used (208, 210).

Phenformin exerts antitumor effects in GSCs overcoming resistance to temozolomide, suppressing GSC self-renewal via the reduction of the expression of stemness and mesenchymal markers, and the increase of miR-124, miR-137 and let-7 expression (221). Phenformin activity has been also analyzed as potential way to disrupt energetic/metabolic pathways sustaining GSC survival and proliferation (222, 223). *In vivo*, phenformin added to the drinking water, caused a significant inhibition of the growth of GBM, in an orthotopic model in which patient-derived GSCs were grafted in nude mice (221), confirming that beside metformin also other biguanides are active against GBM.

In fact, biguanides, unrelated to the antidiabetic drugs (i.e., moroxydine, and cycloguanil) were also reported to significantly reduce proliferation, self-renewal and invasiveness of GSCs, showing higher *in vitro* potency than metformin (211). This observation suggests that antitumor activity against the GBM stem cell-like compartment is a common feature of all biguanides. However, if this is true, all the biguanide moiety-containing molecules have to act through a common intracellular mechanism and all the other pathways proposed for metformin antitumor activity should represent tumor-specific downstream effectors dependent on a common effector which represents the direct biguanide target.

### CLIC1 as Preferential Molecular Target Mediating Metformin, and Other Biguanides, Antitumor Effects in Glioblastoma Stem Cells

The controversies about the anticancer mechanisms of metformin led to overemphasize the role of AMPK in GSC antiproliferative effects, since the liver anti-hyperglycemic activity of this drug is mediated through the activation of this kinase (142). However, a growing bulk of evidence reported, in different cancer models, and in CSCs in particular, that (i) several AMPK-independent pathways are activated by metformin (70, 217); (ii) contrarily to what initially hypothesized, AMPK agonists enhance cancer cell proliferation and metabolism under metabolic stress (i.e., A-769662), while metformin and phenformin inhibit these cellular functions in an AMPK-independent manner (224); (iii) other compounds with a biguanide structure (i.e., moroxydine and cycloguanil), used with different clinical indications, and devoid of AMPK-related effects in the liver, induce the same anti-proliferative and anti-invasive activity in GSCs (211). The latter evidence strongly supports the possibility that, in GSCs, a common molecular target can be hit by all the biguanide-based compounds representing a new pharmacological class effect.

In this line of research, the observation that metformin and related compounds exert their activity on CSCs, not only in GBM (70, 208, 210, 211, 221) but also in different tumor types, such as breast cancer (121), while differentiated cells composing the tumor mass are relatively spared, clearly indicates that biguanides should interact with a CSC-specific target. In recent years, among the possible cancer-specific molecular targets for metformin, CLIC1 has been proposed to represent the main transducer of the biguanide effects in GSCs (68, 211). As detailed in the previous paragraphs, CLIC1 behaves as CSC-specific target because, although expressed in most normal and differentiated (non-stem) tumor cells, it is mainly present as inactive cytosolic monomer, with a very low activation rate (68, 211). This activation kinetics renders non-CSC subpopulations (and normal cells) relatively independent from CLIC1 for proliferation and survival. Conversely, CLIC1 is functionally expressed in GSCs, where it shows a constitutive activity with a peak at the G1-S transition (67), and its activity is absolutely necessary for GSC proliferation (Figure 2). Metformin treatment causes a significant inhibition of CLIC1 activity, measured by voltage-clamp electrophysiology experiments (Figure 4), reaching at high concentrations (5–10 mM) the same efficacy observed using IAA94. Electrophysiology experiments showed that metformin perfusion decreases the whole cell current that cannot be further reduced by the perfusion of IAA94. Current/voltage relationships show that the current amplitudes, at different membrane potentials, are superimposed, suggesting that the two drugs converge on the same molecular target (68). By single amino acid mutation experiments, metformin was also shown to directly interact with tmCLIC1 through Arg29 located within the inner side of the pore structure of the channel (68) (Figure 2). Interestingly this binding site is different from that of IAA94 identified as the external Cys24 (35) allowing a possible discrimination between the effects of the two drugs.

CLIC1 blockade directly correlates with the antiproliferative effects of metformin causing GSC arrest in the G1 phase of the cell cycle. Conversely, metformin, used at the same concentrations, was harmless for cells in which CLIC1 activity was negligible (i.e., MSCs or differentiated GBM cells) confirming the specificity of these effects for GCSs (Figure 4). Moreover, the down-regulation of CLIC1, while reducing the growth rate of GSCs (30) also diminished the antiproliferative activity of metformin, corroborating the hypothesis that, at least in these cells, CLIC1 is the main target of this biguanide (68, 211). This evidence implies that, although metformin directly or indirectly modulates different intracellular signaling, the inhibition of CLIC1 activity is sufficient and necessary to induce antiproliferative activity, at least in GSCs. Moreover, it is important to remark that the inhibition of a GSC-specific molecular target (i.e., tmCLIC1) confers metformin with high selectivity against tumor cells, while sparing normal cells, as also confirmed by the known very low toxicity observed when metformin is used as antidiabetic agent. This observation provides the molecular basis for metformin repositioning as promising novel antitumor agent, being at the same time highly effective toward tumor cells and causing low systemic toxicity (78).

A main issue in metformin-induced tmCLIC1 blockade (and in its antitumor activity, in all the tumor models analyzed) is the high drug concentration (up to 10 mM) required to induce an effect.

Thus, a potential new a therapy could be really successful if retains the efficacy and the discrimination capability among healthy and cancer cells, provided by CLIC1 localization, and the ability to block tmCLIC1, as metformin, but acting at lower doses. The search for novel, more potent tmCLIC1 inhibitors can have a big advantage whether the channel targeting ability can be shared by different structurally-related molecules representing a pharmacological class effect for biguanides. In this line, it is relevant that also phenformin and buformin, former antidiabetic biguanides, were reported to behave as anti-tumor agents (114).

The possible role of CLIC1 as molecular determinant of biguanide antitumor class effect has been recently analyzed in several patient-derived GSC cultures (211). In this study, metformin and phenformin, representative of the antidiabetic biguanides for which antitumoral activity was already proposed, were compared with two antimalarial compounds, proguanil and cycloguanil, and the antiviral compound moroxydine. All these molecules inhibited GSC proliferation, self-renewal, migration and invasion, showing a much higher potency that metformin (up to 50 fold lower IC_50_ than metformin observed with cycloguanil). However, proguanil effects were not specific since it was similarly toxic for GSC and normal stem cells. The direct effects of these molecules on CLIC1 activity were measured by electrophysiology experiments. All the compounds, but proguanil, exerted a significant inhibition of CLIC1-dependent ion current, acting at potency and efficacy related to the respective antiproliferative activity. The lack of efficacy of proguanil as far as CLIC1 inhibition, was proposed to be dependent on the simultaneous presence of the 1-(4-chlorophenyl) ring and the bulky 5-isopropyl group on the rigid biguanide skeleton, thus preventing the access to CLIC1 pore region.

Evidence from this study strengthen the hypothesis that molecules containing a biguanide moiety are potent CLIC1 inhibitors and, consequently, drugs able to selectively interfere with GSC proliferation, migration and self-renewal. Importantly, higher potency than metformin on both cell proliferation and CLIC1 activity inhibition was also demonstrated by these biguanides, suggesting that more easily reachable concentrations of these drugs could be similarly active as the high doses of metformin. Although all these drugs have known limitation for chronic use in patients with GBM, these data demonstrated that CLIC1 inhibition is not only a pharmacological property of metformin, but it may represent a class effect endowed of all the compounds containing a biguanide structure. The relevance of this information resides in the possibility to develop novel biguanide containing drugs, which retain the safety profile of metformin but endowed with increased efficacy and potency toward GSCs.

## Conclusions and Future Presepectives

At odd with most malignant tumors, therapeutic perspective for GBM did not significantly progress in the last decades. In this context, GSCs play a central role in drug resistance, being still extremely elusive as far biological features and pharmacological sensitivity. However, the recent identification that CLIC1 activity is necessary for GSC proliferation, self-renewal and invasiveness, while it is dispensable for most non-transformed normal cell populations, opened new perspectives in the potential development of new therapeutics for this still incurable tumor. This observation found new strength after the recent report that metformin is an efficient inhibitor of CLIC1 activity, although with low potency (IC_50_: 10–30 mM) (211). These data are extremely relevant due to the strong interest in metformin repositioning as antitumoral agent. Several epidemiological, preclinical, and, more recently, some clinical trials are addressing the efficacy of this biguanide in basically all the human tumor types. Conversely, pharmacokinetic and even pharmacodynamic issues are still unsolved to better translate this information in a clinical setting. In particular, as far as GBM is concerned, the main intracellular mechanism associated to metformin antiproliferative activity, the activation of AMPK and the consequent mTOR inhibition, had to be discarded since AMPK was discovered to promote GSC proliferation. Thus, metformin antiproliferative activity has to depend on a completely different mechanism from its glucose-lowering effects. Among all the reported intracellular pathways affected by metformin in tumor cells, the inhibition of CLIC1 activity is of particular interest since it is GSC-specific (thus its targeting does not affect normal cell viability), in line with the low toxicity of the drug when chronically used in T2D patients. Moreover, this effect was directly evaluated by electrophysiology measurement preventing the possibility of effects mediated indirectly by other biochemical regulators. This observation supports that, in GSCs, the inhibition of CLIC1 is a common effect different drugs containing a biguanide structure.

In conclusion, the inhibition of CLIC1 is a novel and unexpected biguanide class effect, which could be used to develop novel drugs with a strong efficacy against GSCs. In fact, although all the biguanides to date tested as inhibitory of CLIC1 activity in GSCs are not completely satisfactory as far as pharmacokinetics and long term tolerability, we believe that this information might pave the way for the identification of novel structurally-related molecules, which in future will provide a better clinical outcome for GBM.

## Author Contributions

All authors listed have made a substantial, direct and intellectual contribution to the work, and approved it for publication.

### Conflict of Interest Statement

The authors declare that the research was conducted in the absence of any commercial or financial relationships that could be construed as a potential conflict of interest.
